# Biallelic variants in *SLC26A2* cause multiple epiphyseal dysplasia-4 by disturbing chondrocyte homeostasis

**DOI:** 10.1186/s13023-024-03228-4

**Published:** 2024-07-02

**Authors:** Shan Li, Yueyang Sheng, Xinyu Wang, Qianqian Wang, Ying Wang, Yanzhuo Zhang, Chengai Wu, Xu Jiang

**Affiliations:** 1 Department of Molecular Orthopaedics, National Center for Orthopaedics, Beijing Research Institute of Traumatology and Orthopaedics, Beijing Jishuitan Hospital, Capital Medical University, Beijing, China; 2 Department of Orthopaedics, National Center for Orthopaedics, Beijing Jishuitan Hospital, Capital Medical University, Beijing, China

**Keywords:** Multiple epiphyseal dysplasia-4 (MED-4), *SLC26A2* gene, Compound heterozygous, Loss-of-function, Cartilage homeostasis

## Abstract

**Background:**

Multiple epiphyseal dysplasia-4 (MED-4, MIM 226900) is a rare autosomal recessive disease characterized by disproportionate height and early onset osteoarthritis of the lower limbs. MED-4 is caused by homozygous or compound heterozygous pathogenic variants in the *SLC26A2* gene. However, the underlying pathogenic mechanisms in chondrocytes remains unknown. This study aimed to identify the pathogenic variants within a MED-4 family and explore the molecular etiology of this condition in human primary chondrocyte cells.

**Methods:**

Clinical data were recorded and peripheral blood samples were collected for analysis. Whole exome sequencing (WES) and bioinformatic analyses were performed to determine causative variants. Wild-type *SLC26A2* and corresponding mutant expression plasmids were constructed and transfected into human primary chondrocytes. The expression and subcellular distribution of SLC26A2 protein in chondrocytes were detected by immunoblotting and immunofluorescence. Effects of these variants on chondrocytes viability and apoptosis were measured by Cell Counting Kit-8 (CCK-8) assay. Expression of genes related to cartilage homeostasis was subsequently analyzed by quantitative real-time polymerase chain reaction (qRT-PCR).

**Results:**

We identified two compound heterozygous variants c.1020_1022delTGT(p.Val341del) and c.1262 T > C(p.Ile421Thr) in the *SLC26A2* gene in the patients. Mutant SLC26A2^Val341del^ and SLC26A2^Ile421Thr^ proteins were distributed in relatively few cells and were observed only within the nucleus. The viability of chondrocytes with the *SLC26A2* variant group was similar to the wild-type (WT) group. However, the protein expressions of SLC26A2^Val341del^ and SLC26A2^Ile421Thr^ were decreased compared with SLC26A2^WT^. Expression levels of matrix metallopeptidase 13 (*MMP13*), α-1 chain of type X collagen *(COL10A1*), and Runt-related transcription factor 2 (*RUNX2*) were significantly decreased in the variant group. However, aggrecan (*ACAN*) expression was higher in the variant group than the WT group.

**Conclusions:**

Overall, our data demonstrate that the variants p.Val341del and p.Ile421Thr in *SLC26A2* cause MED-4 and that these two variants promote chondrocyte proliferation while inhibiting chondrocyte differentiation.

**Supplementary Information:**

The online version contains supplementary material available at 10.1186/s13023-024-03228-4.

## Introduction

Multiple epiphyseal dysplasia (MED) is a genetically and clinically heterogeneous epiphyses dysplasias characterized by short stature, arthralgia, joint deformities, abnormal gait, and early-onset osteoarthritis [1]. Some patients with MED are born without abnormalities but exhibit slow height growth during childhood, eventually leading to moderate short height [1, 2]. Approximately half of affected individuals have an abnormal finding at birth, such as clubfoot, clinodactyly, or cystic ear swelling (rarely). Onset of articular pain is variable, but commonly occurs in late childhood [2]. Before puberty, stature is usually within the normal range; in adulthood, stature is only mildly diminished, ranging from 150 to 180 cm [1, 2]. During childhood and adolescence, functional disability is mild or absent. Joint involvement progresses slowly in young adults, and hip and knee surgery is usually unnecessary [2]. In 1947, Fairbank first described the clinical features of MED through radiographic examination. The radiographs showed structural anomalies in epiphyses and delayed ossification of the epiphyses with small, irregular ossification centers [3]. On lateral knee radiographs, a double-layered patella is a quite specific sign of MED causing by *SLC26A2* in approximately 60% of individuals [2]. To date, variants in seven genes: cartilage oligomeric matrix protein (*COMP*, MIM 600310), matriline-3 (*MATN3*, MIM 602109), α 1–3 chains of type IX collagen (*COL9A1*, MIM 120210; *COL9A2*, MIM 120260; *COL9A3*, MIM 120270), sulfate transporter (*SLC26A2*, MIM 606718), and calcium-activated nucleotidase-1 (*CANT1*, MIM 613165), have been found to be responsible for MED [4–8]. The first five exhibit autosomal dominant inheritance, while the *SLC26A2* is autosomal recessive [1].

The genetic variants in *SLC26A2* result in skeletal dysplasias, which range in severity from the perinatal lethal achondrogenesis type IB (ACG1B, MIM 600972), atelosteogenesis type II (AO2, MIM 256050), and nonlethal diastrophic dysplasia (DTD, MIM 222600) to the relatively mild recessive multiple epiphyseal dysplasia-4 (MED-4, MIM 226900) [9–18]. *SLC26A2* (NM_000112.4) is located on chromosome 5q32 and consists of 2 exons, encoding an 82 kDa protein with 739 amino acids. Transmembrane protein SLC26A2 functions as a plasma membrane sulfate/chloride antiporter, responsible for inorganic sulfate uptake and proteoglycan sulfonation [19]. *SLC26A2* is essential for the process of endochondral ossification and maintaining the sulfation of cartilage matrix proteoglycans. It has recently been demonstrated that the Slc26a2 knock-out mouse model of ACG1B and AO2 has overactivated fibroblast growth factor receptor 3 (FGFR3) signaling [20]. Chondrocytes in Slc26a2^−/−^ mice have impaired extracellular deposition of collagen ColII and ColIX, leading to defective cartilage formation [20]. Park et al. reported that *SLC26A2* regulates chondrocyte, proliferation, differentiation and proteoglycan synthesis activated by IGF-1 [21]. Previous studies have shown that the clinical severity of the phenotypes depend on the residual activity of sulfate transporter [20]. However, recent research suggests that the correlation between residual sulfate uptake function, proteoglycan undersulfation, and clinical severity of *SLC26A2*-associated skeletal diseases is not absolute. This indicates that other unknown factors may contribute to and modify the severity of phenotypes [22].

In this study, we investigated two patients from a Chinese family who presented with scoliosis, short stature, spinal stenosis, and congenital bilateral equine varus with epiphyseal dysplasia. Using whole-exome and Sanger sequencing, we identified compound heterozygote variants c.1262 T > C(p.Ile421Thr) and c.1020_1022delTGT(p.Val341del) in the *SLC26A2* gene of these patients. In addition, we utilized in silico and in vitro techniques to confirm the pathogenicity of these variants. Based on these findings, we propose a molecular mechanism to explain the impact of *SLC26A2* variants on human primary cartilage homeostasis.

## Materials and methods

### Blood sample collection

Informed written consents for the genetic analysis and publication of data were obtained from three adult participants. This study was performed in accordance with the Helsinki Declaration and was approved by the Ethics Committee of the Beijing Jishuitan Hospital affiliated with Capital Medical University. 5 ml peripheral blood samples were collected using EDTA tubes. Genomic DNA was isolated using the QIAamp DNA Blood Midi Kit (Qiagen, Hilden, Germany) and quantified by the Nanodrop 2000 spectrophotometer (Thermo Scientific).

### Whole exome sequencing (WES) and data analysis

Whole-exome sequencing (WES) was performed in the proband (II-1) and her family members (II-3, I-1). Sequencing libraries were generated by the Agilent SureSelect Human All Exon V6 kit (Agilent Technologies, USA), and subsequently sequenced on the HiSeq 2000 platform (Illumina, San Diego, CA). The initial fluorescence image files underwent base calling to convert them into short reads (Raw data). Raw data containing adapator and low-quality reads were filtered to obtain clean data [23]. Burrows-Wheeler Aligner software mapped clean reads mapped to the human reference genome sequence (GRCh37/hg19). WES quality control data is illustrated in Table 1.

**Table 1:** Quality control data of whole exome sequencing of three samples

Sample	I1	II1	II3
Total	87,585,142 (100%)	109,546,006 (100%)	94,149,168 (100%)
Duplicate	21,017,198 (24.06%)	27,390,831 (25.06%)	21,137,981 (22.50%)
Mapped	87,340,670 (99.72%)	109,291,245 (99.77%)	93,949,877 (99.79%)
Properly mapped	86,410,904 (98.66%)	108,529,926 (99.07%)	93,112,880 (98.90%)
PE mapped	87,214,148 (99.58%)	109,159,474 (99.65%)	93,841,922 (99.67%)
SE mapped	253,044 (0.29%)	263,542 (0.24%)	215,910 (0.23%)
Raw reads	47,913,036	58,922,788	52,301,034
Raw data(G)	14.37	17.68	15.69
Raw depth(x)	237.69	292.44	259.52
Effective(%)	91.40	92.96	90.01
Error(%)	0.03	0.03	0.03
Q20(%)	97.17	97.27	97.38
Q30(%)	92.74	92.96	93.2
GC(%)	49.17	50.44	50.75
Initial_bases_on_target	60,456,963	60,456,963	60,456,963
Initial_bases_on_or_near_target	136,297,444	136,297,444	136,297,444
Total_effective_yield(Mb)	13,033.29	16,307.88	14,024.63
Effective_yield_on_target(Mb)	6,916.09	8,416.91	7,973.69
Fraction_of_effective_bases_on_target	53.1%	51.6%	56.9%
Fraction_of_effective_bases_on_or_near_target	74.7%	74.1%	78.1%
Average_sequencing_depth_on_target	114.40	139.22	131.89
Bases_covered_on_target	60,381,182	60,252,533	60,384,309
Coverage_of_target_region	99.9%	99.7%	99.9%
Fraction_of_target_covered_with_at_least_100x	48.5%	58.3%	55.1%
Fraction_of_target_covered_with_at_least_50x	86.5%	91.0%	89.4%
Fraction_of_target_covered_with_at_least_20x	98.0%	98.7%	98.7%
Fraction_of_target_covered_with_at_least_10x	99.3%	99.3%	99.5%
Fraction_of_target_covered_with_at_least_4x	99.7%	99.6%	99.8%

Variants were filtered with minor allele frequency (MAF) < 1% in the frequency databases, including dbSNPs, gnomAD, 1000 Genomes database, Exome ExAC and esp6500si_all. The previous literature and Human Gene Mutation Database (HGMD) were used to determine if the variants were novel or known. Mutation Taster, Polyphen-2, and M-CAP were then used to predict the pathogenicity of these variants. Finally, the pathogenicity of variants was assessed using the 2015 criteria of American College of Medical Genetics and Genomics (ACMG). Meanwhile, we compared the variants found in patient and other affected or unaffected family members [24]. The OMIM database and previously published articles have been used to establish gene function. The method used was similar to that used in our previous study [25]. The detailed variant interpretation and analysis process is presented in Figure S1.

### Sanger sequencing and T-clone sequencing

To validate the candidate variants c.1262 T > C and c.1020_1022delTGT in *SLC26A2*, sequences were amplified by PCR followed by Sanger sequencing. Due to the close proximity of the two variants, the missense c.1262 T > C variant was buried in the mantle of the deletion variant. Sanger sequencing of the patients results showed interlaced alleles. The purified PCR products with disrupted signals were linked to the pMD19-T vector to perform T-Clone sequencing. The method used was similar to that used in our previous study [26]. All primers were designed using the online tool Primer3 (Table S1).

### Molecular modeling and structural analysis

The amino acid sequences of the SLC26A2 were obtained from the NCBI Protein database (FASTA format). Multiple sequence alignments with different species and conservative analysis were performed using the software MEGA. 3D structures of normal and missense variant in the SLC26A2 protein were generated by homology modeling using the SWISS-MODEL server. The interactions between amino acid and adjacent residues were simulated with the PyMOL (Schrödinger, LLC).

### Plasmids construction and transfection

The wild type of pcDNA3.1( +)-SLC26A2 plasmids were purchased from nuosai (Beijing, China). The mutant expression vectors of Val341del and Ile421Thr were constructed using pcDNA3.1-SLC26A2 as the template for mutagenesis by PCR with mutagenic primers, and the variants were identified by Sanger sequencing. Purchased commercial human primary cartilage cells (Bena Culture Collection, China) were cultured in Dulbecco’s modified Eagle’s Medium (DMEM, Gibco) containing 10% fetal bovine serum (FBS, Gibico), L-glutamine and 1% penicillin–streptomycin at 37 °C and at 5% CO_2_ atmosphere. The human primary cartilage cells transfected with pcDNA3.1( +) plasmids containing the wild-type *SLC26A2* gene and the mutated *SLC26A2* (c.1020_1022del and c.1262 T > C) using Lipofectamine 3000 (Invitrogen, USA), were named WT and MUT, respectively. Additionally, cells transfected with the pcDNA3.1( +) plasmid were used as a control.

### Immunofluorescence staining

After 48 h of transfection, cells were fixed with 4% paraformaldehyde, permeabilized with 0.2% Triton X-100 for 20 min, and then blocked with 1% BSA in PBS for 60 min. Subsequently, the cells were stained with primary monoclonal anti-SLC26A2 antibody (Thermo-PA576918, 1:200) and incubated at 37 °C for two hours. Alexa Fluor 488 conjugated anti-rabbit IgG secondary antibodies (Abcam-ab150077, 1:400) were incubated at room temperature without light for 60 min. After washing 3 times with PBS, DAPI nuclear staining was added for 5 min. Slides were imaged using an OLYMPUS fluorescence microscope with a 20 × objective lens.

### Cell viability assay

Cells were transfected by four different plasmids (pcDNA3.1, pcDNA3.1-SLC26A2^WT^, pcDNA3.1-SLC26A2^Val341del^ and pcDNA3.1-SLC26A2^Ile421Thr^ as described above. The viability of chondrocyte cells at different time points was determined using the Cell Counting Kit-8 (CCK-8) assay (Dojindo Molecular Technologies, Tokyo, Japan). Briefly, 10 μl CCK-8 solution was mixed to the 100 μl cells medium. Then, 100 μl of CCK-8 medium was added to the cells and incubated at 37 °C for two hours. The absorbance values at 450 nm were detected using a microplate spectrophotometer.

### Quantitative real-time PCR

Total RNA was extracted using Trizol (Invitrogen, USA) and reverse transcribed into cDNA templates using the RT reagent Kit with gDNA Eraser (RR047A, Takara Bio), according to manufacturer protocol. Primer sequences used for amplification are listed in Table S1. The cDNA was amplified with 40 reaction cycles for sequencing analysis. The relative levels of messenger RNA (mRNA) for the genes of interest were normalized to GAPDH.

### Western blotting analysis

Chondrocytes were lysed and centrifuged at 12,000xg at 4 °C for 10 min to collect total protein. Equal amounts of protein (25 μg) from different samples were separated by SDS-PAGE using a 4%-12% gradient gel, and then transferred onto a PVDF membrane. Membranes were then incubated with primary antibodies against SLC26A2 (1:1000, Thermo-PA576918) and β-actin (1:1000, Abcam-ab8226) at 4 °C overnight. HRP-conjugated anti-rabbit IgG antibody (1:1000, CST) or goat anti-mouse IgG (1:1000, Biorigin-BN20604) was added, followed by detection using ECL Plus reagent (Millipore). The images were recorded using a Gel Imaging System (Bio-Rad Laboratories, USA).

### Statistical analysis

All data are presented as mean ± SD. One-way analysis of variance (ANOVA) was used to evaluate the differences among groups. All statistical analyses were performed using GraphPad Prism version 9.0 (GraphPad Software, Inc.). *P*-values of < 0.05 were considered statistically significant.

## Results

### Clinical presentation

**Fig. 1:**
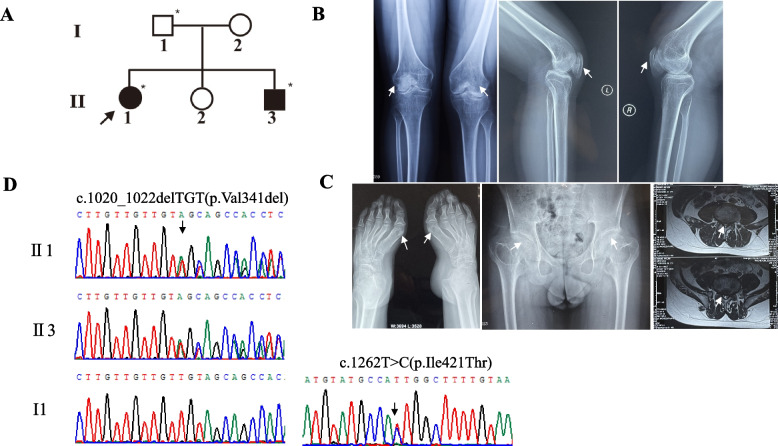
Pedigree, clinical features and identification of pathogenic variants in patients. **A** Black arrow denotes the proband. Open circles and squares represent unaffected females and males. Black-filled circles and squares represent affected members. **B** X-ray image of the distal femur and proximal fibular tibia of Patient 1(II1). **C** Clinical presentation of Patient 2 (II3) showing congenital bilateral clubfoot equine, hip epiphyseal dysplasia and spinal stenosis. **D** Sequence chromatograms from Sanger sequencing analysis of the *SLC26A2* gene showing the compound heterozygous pathogenic variants c.1020_1022del and c.1262 T > C in two patients and a heterozygous state in their father

Two Chinese patients (II1, II3) were born to parents nonconsanguineous who were phenotypically normal (Fig. 1A). The clinical symptoms of patient 1(II1) (34 years old, height 141 cm, Z-score = -3.86) are small stature, scoliosis, and necrosis of the femoral head (Fig. 1B). X-ray of patient II1 show bilateral layered patella. Also, the shape of tibia and femoral diaphysis is not regular, and the bone density is not uniform (Fig. 1B). Patient 2 (II3) (23 years old, height 165 cm, Z-score = -1.36) exhibit clinical presentations including spinal stenosis, congenital bilateral clubfoot equine. X-ray of patient II3 show that metatarsal bones are short and thick, and the metaphyseal is irregular and enlarged (Fig. 1C). Bilateral hip acetabular dysplasia, femoral head flattened, femoral neck widened, bilateral femoral shaft thinner are shown in Fig. 1C. Clinical symptoms of the two patients are shown in Table 2.

**Table 2:** Summary of clinical symptoms of the two patients

Clinical phenotypes	Patient (II1)	Patient (II3)
Sex	Female	Male
Age	34 years	23 years
Small femoral heads	ND	+
Short stature	+	+
Scoliosis	+	-
Hip dysplasia	+	+
Flattened proximal femoral epiphyses	NA	+
Limited elbow flexion	+	+
Double layered patella	+	ND
Arthralgia	+	+
Mild frontal hypoplasia	NA	NA
Flat proximal femoral epiphyses	NA	+
Spinal stenosis	-	+
Brachydactyly	+	+
Mild shortened metacarpals	+	+
Clubfoot	-	+

“ + ”, present, “-”, absent, “*NA*”, information not available, “*ND*”, not determined

### Identified compound heterozygous variant in SLC26A2

**Fig. 2:**
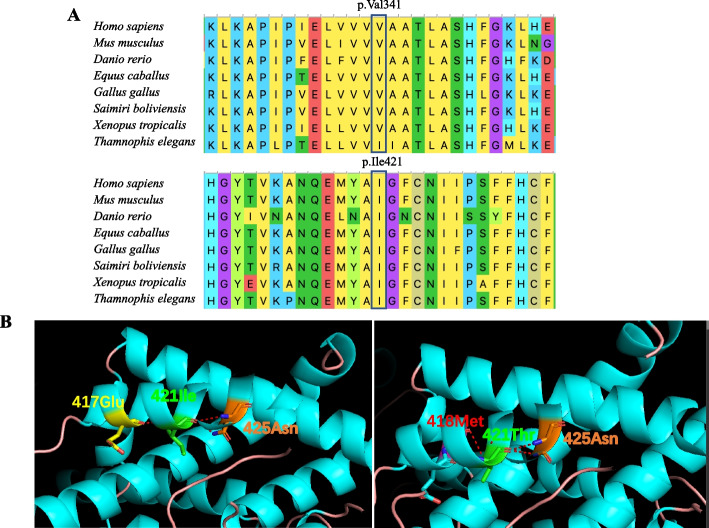
Characterization of identified variants in *SLC26A2*. **A** Conservation analysis of Val341 and Ile421 residues (highlighted in box) in *SLC26A2* across different species. **B** Three-dimensional structure of the missense variant site Ile421 in *SLC26A2*. A hydrogen bond between Ile421 and Glu417 is disrupted by the variant p.Ile421Thr

Based on the inheritance patterns, Table S2 show rare variants shared by the two affected subjects. During analysis of rare variant association based on phenotype, rare variants related to bone development found in patients are listed in the Table S3. Bioinformatics analysis revealed two likely pathogenic variants in the *SLC26A2* gene in both two patients. The patient’s DNA amplified with the primer of SLC26A2-E2-F/R primers (Table S1) combined with T-cloning analysis confirmed that patients had c.1020_1022delTGT and c.1262 T > C variants in exon 2 (Figure S2, Fig. 1D). A known heterozygous frameshift variant, c.1020_1022delTGT(p.Val341del), was identified, resulting in the deletion of valine at codon 341 (Fig. 1D). The frequencies of this variant were reported to be 0.014% in ExAC and 0.018% in gnomAD_ ALL. However, this variant was not detected in the 1000 g or 1000g_EAS. A novel missense variant of paternal origin c.1262 T > C (Fig. 2) was also identified. The frequency of this variant is reported to be 0.0008% in ExAC and was not reported in gnomAD_ ALL. Frequencies of these two variants reported in the genetic databases are summarized in Table S4.

### Bioinformatics analysis of the variants

Conservation analysis indicated that amino acid sites p.V341 and p.I421 of the solute carrier family 26 member 2 protein are highly conserved (Fig. 2A), suggesting their crucial functional role in growth and development. The missense mutant SLC26A2 protein showed the structural alteration (Fig. 2B). In the wild-type protein, Ile421 is predicted to form hydrogen bonds with 417Glu and 425Asn. However, the variant causes steric hindrance, resulting in hydrogen bonds at 418Met instead of 417Glu, potentially altering its biological function (Fig. 2B). The Bioinformatic analysis provided additional insights into the possible pathogenicity of the variants.

### Cellular distribution and functional expression of SLC26A2 were changed

**Fig. 3:**
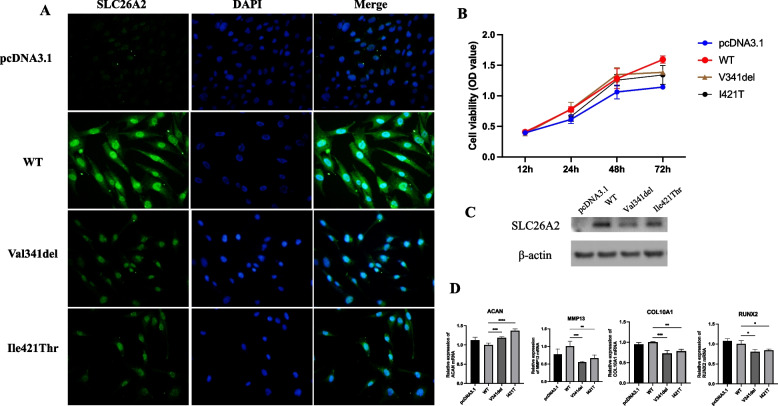
Function of *SLC26A2* variants p.Val341del and p.Ile421Thr. **A** Cellular localization of SLC26A2-WT, SLC26A2-Val341del and SLC26A2- Ile421Thr in cells transfected with plasmids pSLC26A2-WT, pSLC26A2-Val341del or pSLC26A2-Ile421Thr. Cells were stained with anti-SLC26A2 antibody. Nuclei were stained with DAPI (Blue). **B** Growth curve analysis of human primary chondrocytes transfected with pSLC26A2-WT, pSLC26A2-Val341del or pSLC26A2-Ile421Thr. Control cells were transfected with pcDNA3.1. **C** The protein expression levels of *SLC26A2* mutants was measured by Western blot. **D** mRNA expression levels were assessed by qRT-PCR. Error bars represent the SD of four independent experiments

We performed immunofluorescent staining and Western blotting assay to determine whether there are differences between the amount and the cellular location of expressed mutant *SLC26A2*. Little immunofluorescence staining was observed in cells transfected with the vector alone. In cells transfected with wild-type *SLC26A2*, immunofluorescence was observed at the plasma membrane of the cell, with additional signal detected in the cytoplasm and nucleus. In contrast, immunofluorescence of the Val341del and Ile421Thr variant was detected in relatively few cells and was primarily localized to the nucleus, as illustrated in Fig. 3A.

To assess the potential impact of the variants on chondrocyte cell viability, we conducted a CCK-8 assay to measure cell proliferation and viability at various time points after transfection with either wild-type or mutant plasmid. We found that cells transfected with the Val341del and Ile421Thr exhibited a similar viability and proliferation rate at hours 24, 48, and 72 to that cells of transfected with wild-type *SLC26A2* (Fig. 3B). As shown in Fig. 3B, the two variants did not appreciably affect cell viability and proliferation. However, Western blot analysis revealed a significant reduction in the relative expression level of SLC26A2 protein in cells carrying the Val341del and Ile421Thr variants compared to wild-type (Fig. 3C). These results suggest that variants affect the subcellular localization and protein expression levels of SLC26A2.

### SLC26A2 variant disrupts the homeostasis of cartilage cells

The results of qRT-PCR indicate a significant decrease in the relative mRNA expression of chondrocyte differentiation genes, such as *COL10A1*, *RUNX2*, and *MMP13*, in the mutant group compared to the wild-type group (Fig. 3D). However, compared to wild-type, relative mRNA expression levels of the anabolic gene *ACAN* in chondrocytes in the mutant group was significantly higher (Fig. 3D). These findings suggest that *SLC26A2* variants disrupt the homeostasis of chondrocyte cells by decreasing chondrocyte differentiation and promoting anabolic processes.

## Discussion

Multiple epiphyseal dysplasia belongs to a group of genetically heterogeneous skeletal dysplasia, with seven pathogenic genes currently known [2]. MED is a heterogeneous group of skeletal dysplasias characterized by dysplastic epiphyses in numerous sites [2, 5]. However, diagnosis requires a combination of clinical and radiological symptoms. Due to various complications and similarities to related diseases, accurate clinical diagnosis of skeletal dysplasias categories is challenging by conventional means and genetic testing plays a critical role [13, 15].

In this study, we reported two patients with epiphyseal dysplasia born to non-consanguineous parents. Patient 1 also presented with scoliosis, clubfoot, double-layered patellae, and aseptic necrosis of the hip joint. Her younger brother, Patient 2, presented with horseshoe varus and spinal stenosis. WES and bioinformatic analysis were conducted and identified compound heterozygous variants (c.1020_1022del; c.1262 T > C) in the *SLC26A2* gene in the affected patients. It is known that c.1262 T > C originated from the father (I1). Simple genetics suggests the c.1020_1022del variant was inherited from the mother (I2). Unfortunately, because of the unavailability of peripheral blood samples from the mother, confirmation of this was not possible. Through species conservation, 3D protein models, bioinformatics analysis tools and co-segregation research, the Ile421Thr missense variant has been shown to have detrimental effects. This variant has been classified as “pathogenic” (PVS1 + PM1 + PM2) according to the ACMG guidelines. Compound heterozygous c.1020_1022del and c.1262 T > C variants in the *SLC26A2* gene were identified to be the cause of MED-4 after conducting a thorough analysis of the clinical manifestations.

The *SLC26A2* gene is associated with four types of autosomal recessive achondrogenesis [16–20], resulting in phenotypes consistent with MED-4. Although MED-4 has been reported in different populations, it appears to be less prevalent among the Chinese population, with only two cases reported to date [10].

**Fig. 4:**
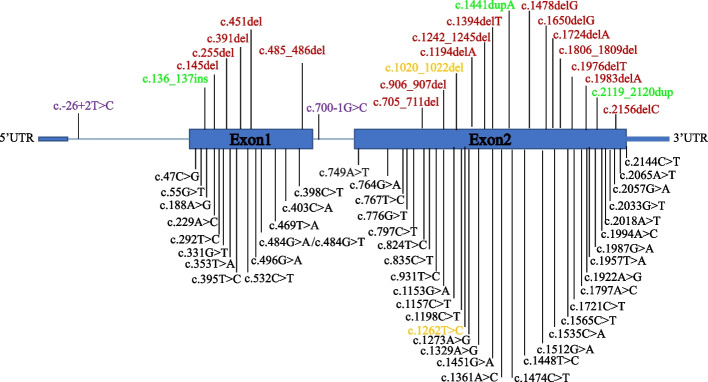
Variant spectrums of the *SLC26A2* gene. Blue boxes represent the exons in each gene, and lines represent the introns. The variants identified in this study work are highlighted in yellow. Missense/nonsense variants are shown in black. Splicing, deletion, and insertion variants are marked in purple, red and green, respectively

To date, 69 variants in the *SLC26A2* gene have been reported in the professional Human Gene Mutation Database (HGMD), including 46 missense/nonsense variants (46/69, 66.67%), 2 splice variants, 18 small deletions and 3 small insertions (Fig. 4, Table S5) [9, 26–35]. The homozygous deletion variant c.1020_1022del(p.Val341del) was first reported to cause ACG-IB by Superti-Furga et al. whereas the discovery of the c.1262 T > C(p.Ile421Thr) is novel [14]. Recently, the variant c.1020_1022del (p.Val341del) was also reported in a 42-year-old woman with diastrophic dysplasia including congenital dislocation of a hip, arthrogryposis, chronic cholecystitis, left ureter contraction, urolithiasis, chronic gastritis and kyphoscoliosis from west Ukraine [36]. All four patients had the same c.1020_1022del variant, but their clinical features differed substantially. Notably, the phenotype of the patient with homozygous deletion variants is more severe than that of patients with missense variants. This suggests, as might be expected, deletion variants have more detrimental effects than missense variants.

Previous reports have suggested that patients with reduced *SLC26A2* expression exhibit a distinct phenotype, whereas ACG-IB is caused by the null pathogenic variants. Genotype–phenotype correlations indicate that the amount of residual activity of the sulfate transporter modulates the phenotype in this spectrum of disorders, which extends from ACG1B to mild MED-4. Unger et al. reported that homozygosity or compound heterozygosity for pathogenic variants predicting stop codons or structural pathogenic variants in transmembrane domains of the sulfate transporter are associated with ACG1B, while variants located in extracellular loops, in the cytoplasmic tail of the protein, or in the regulatory 5’-flanking region of the gene result in less severe phenotypes [2]. However, in this study, phenotypic differences have been observed between unrelated and even first-degree relatives with the same pathogenic variants, suggests that additional as yet unknown factors influence phenotype severity. Thus, the specific disease indicated by the patients is determined by the expression level of *SLC26A2*.

The effect of *SLC26A2* variants on chondrocytes has not previously been studied to the best of our knowledge. However, several studies have reported a decrease in the rate of sulfate transport and the amount of protein expression of the mutated *SLC26A2* compared to wild-type in *Xenopus* oocytes and mammalian HEK-293 cells [19]. In this study, cell viability did not significantly decrease due to the presence of variants. The variant in *SLC26A2* (c.1262 T > C) results in the amino acid sequence (p.Ile421Thr) and does not cause premature termination of translation. Additionally, as shown in Fig. 2D, Ile421Thr showed slightly elevated relative expression compared to Val341del. The type of variant clearly influences the relative amount of residual protein function, but it is unclear whether the reduced quantity of expressed protein represents decreased synthesis or increased turnover of the mutated protein [19]. Our study also indicates that the *SLC26A2* variant promotes chondrocyte proliferation while inhibiting chondrocyte differentiation. Chondrocytes carrying the *SLC26A2* variant exhibited a higher proliferative rate than wild-type cells. Expression of markers of chondrocyte proliferation was significantly increased in chondrocytes with the *SLC26A2* variant; in contrast, the expression of markers associated with differentiation, including *MMP13*, *COL10A1*, and *RUNX2*, was significantly decreased in chondrocytes with the *SLC26A2* variant. These results may be attributed to the reduced SO_4_^2−^ uptake in cells carrying the *SLC26A2* variant [19]. SO_4_^2−^ plays an important role in chondrocyte proliferation and differentiation, and these effects are mediated by sodium-dependent transporters. It may be that the multiple roles of sulfation contribute to* SLC26A2* mediating a variety of chondrocyte functions [21]. It has also been shown that the ablation of *SLC26A2* in osteoblasts causes severe bone loss and elicits peculiar pericellular matrix (PCM) production characterized by undersulfation coupled with decreased stiffness [37].

It should be noted that there are some limitations in the present study. We used cell models and lacked in vivo animal experiments to investigate the effect of *SLC26A2* variant on chondrocyte proliferation and differentiation. By overexpressing the wild-type and mutant groups, all have a similar baseline level of *SLC26A2* expression, making them comparable.

These findings suggested that variants in *SLC26A2* do not significantly affect significant changes in the viability of chondrocytes but lead to decreased *SLC26A2* expression. *SLC26A2* variants also promotes chondrocyte proliferation while inhibiting differentiation. This study provides new insight to investigate the mechanism of development of multiple epiphyseal dysplasia, whereby variant of *SLC26A2* may contribute to the development of MED-4 by disturbing chondrocyte homeostasis, specifically by inducing disturbance of proliferation and differentiation.

## Conclusion

In conclusion, our study reported two variants, c.1020_1022del and c.1262 T > C in the *SLC26A2* gene associated with multiple epiphyseal dysplasia in a Chinese family. We demonstrated the pathogenicity of c.1262 T > C variant and thereby contributed to a deeper understanding of chondrocyte homeostasis as it relates to MED-4. These findings provide valuable insights into the genetic basis of MED-4 and pave the way for further research in this field.

### Supplementary Information

Additional file 1: Figure S1. Data interpretation pipeline for whole exome sequencing. Figure S2. T-cloning sequencing results of PCR products extracted from the patient’s blood. Sequence analysis confirmed that the c.1262T>C(p.Ile421Thr) variant is present in the disrupted signal caused by the c.1020_1022delTGT(p.Val341del) variant.

Additional file 2: Table S1. Primers used for variants amplification and qPCR.

Additional file 3: Table S2. Rare variants shared by the two affected subjects.

Additional file 4: Table S3. Rare variants related to bone development found in patients.

Additional file 5: Table S4. Pathogenicity prediction of two variants using bioinformatics tools.

Additional file 6: Table S5. List of *SLCA26A2* variants in this study and reported in the literatures.

## Data Availability

The datasets generated for this study are available in online repositories. The names of the repository/repositories and accession number(s) can be found at: https://www.ncbi.nlm.nih.gov/genbank/, NM_000112.4.
