# Supplementary figures and images for: Biallelic variants in SLC26A2 cause multiple epiphyseal dysplasia-4 by disturbing chondrocyte homeostasis
Source: Orphanet J Rare Dis. 2024 Jul 2;19:245. doi: 10.1186/s13023-024-03228-4 (PMC11220988; doi:10.1186/s13023-024-03228-4)

**Figure. S1**

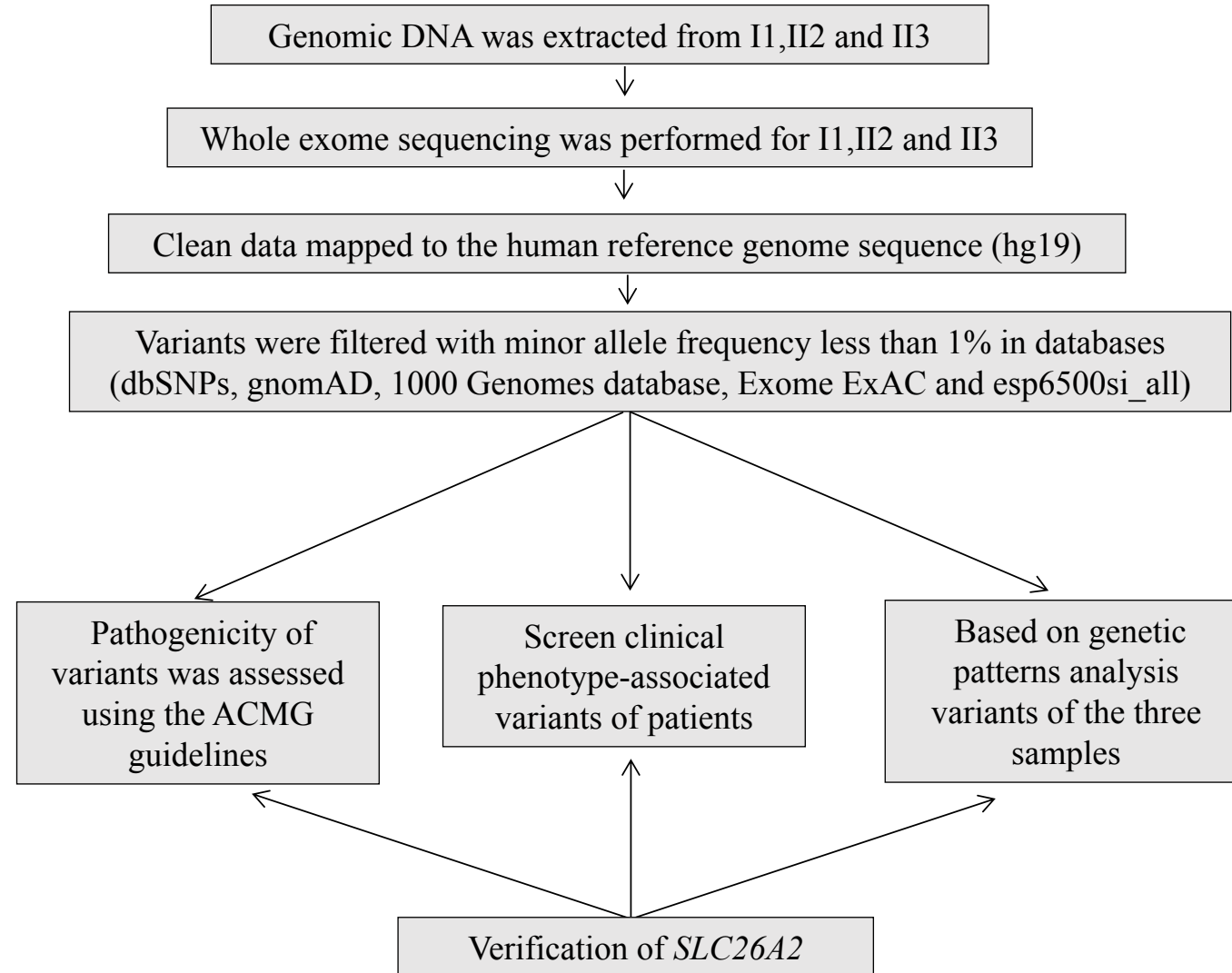

**Figure. S2**

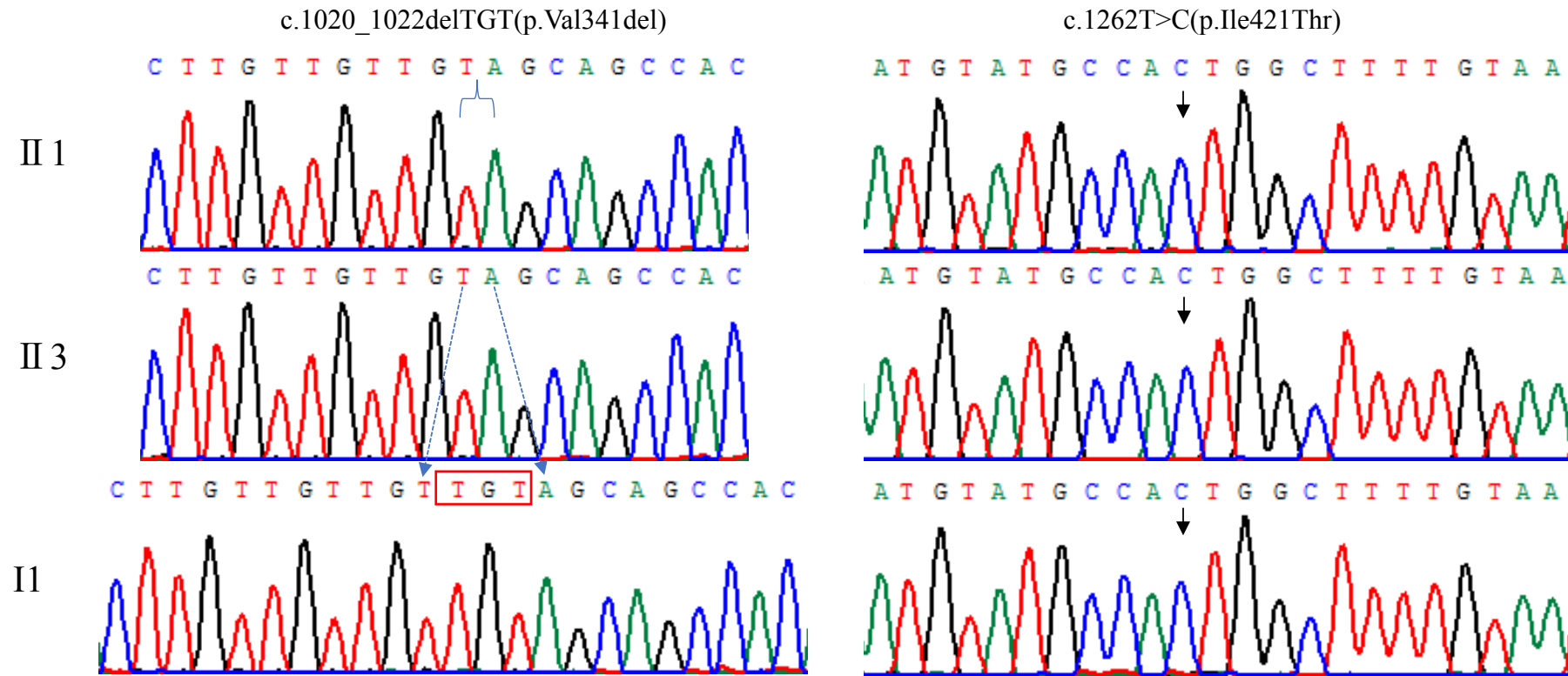

Supplement: Supplementary file 1 — Additional file 1: Figure S1. Data interpretation pipeline for whole exome sequencing. Figure S2. T-cloning sequencing results of PCR products extracted from the patient’s blood. Sequence analysis confirmed that the c.1262T>C(p.Ile421Thr) variant is present in the disrupted signal caused by the c.1020_1022delTGT(p.Val341del) variant. [file 13023_2024_3228_MOESM1_ESM.pdf]
